# Caries in the infundibulum of the second upper premolar tooth in the horse

**DOI:** 10.1186/1751-0147-49-10

**Published:** 2007-03-28

**Authors:** Torbjörn S Lundström, Gunnar G Dahlén, Ove S Wattle

**Affiliations:** 1 Section of Large Animal Medicine and Surgery, Department of Clinical Sciences, Swedish University of Agricultural Sciences, Box 7054, 750 07 Uppsala, Sweden; 2 Laboratory for Oral Microbiology, Faculty of Odontology, Sahlgrenska Academy at Göteborg, Sweden

## Abstract

**Background:**

Swedish equine dental practices have empirically found that the prevalence of infundibular caries as a primary disorder in the first permanent premolar teeth (P2) of the horse upper jaw has increased during the last 10 years. A previously unknown bacterial species, *Streptococcus devriesei *(CCUG 47155^T^), which is related to *Streptococcus mutans*, has recently been isolated from these carious lesions. To understand the aetiology of caries in horses, it is essential to elucidate the relationship between *S. devriesei *and P2 infundibular caries.

**Methods:**

The anterior infundibulum of maxillary P2, or the occlusal surface at the site of the infundibulum, in 117 horses and ponies, 77 with and 40 without caries in this tooth, was sampled for bacteriological analyses between 1990 and 2004. Samples were transported in VMGA III medium and then inoculated onto MSB agar. The approximate number of bacteria was counted in each sample and the isolates were characterised biochemically, using a commercial kit.

**Results:**

All 50 samples taken from carious lesions after 2002 were positive for an *S. mutans*-like strain, i.e. *S. devriesei*. The bacteria were also found in four of the control animals, but were much less numerous than in samples from caries-affected horses. None of the swabs taken prior to 2002 were positive for this bacteria.

**Conclusion:**

Our results demonstrate that *S. devriesei *can colonise the infundibulum of P2 of the horse upper jaw, which can be fatal for the dental tissue. We conclude that *S. devriesei *is strongly associated with P2 caries in horses.

## Background

**Figure 1:**
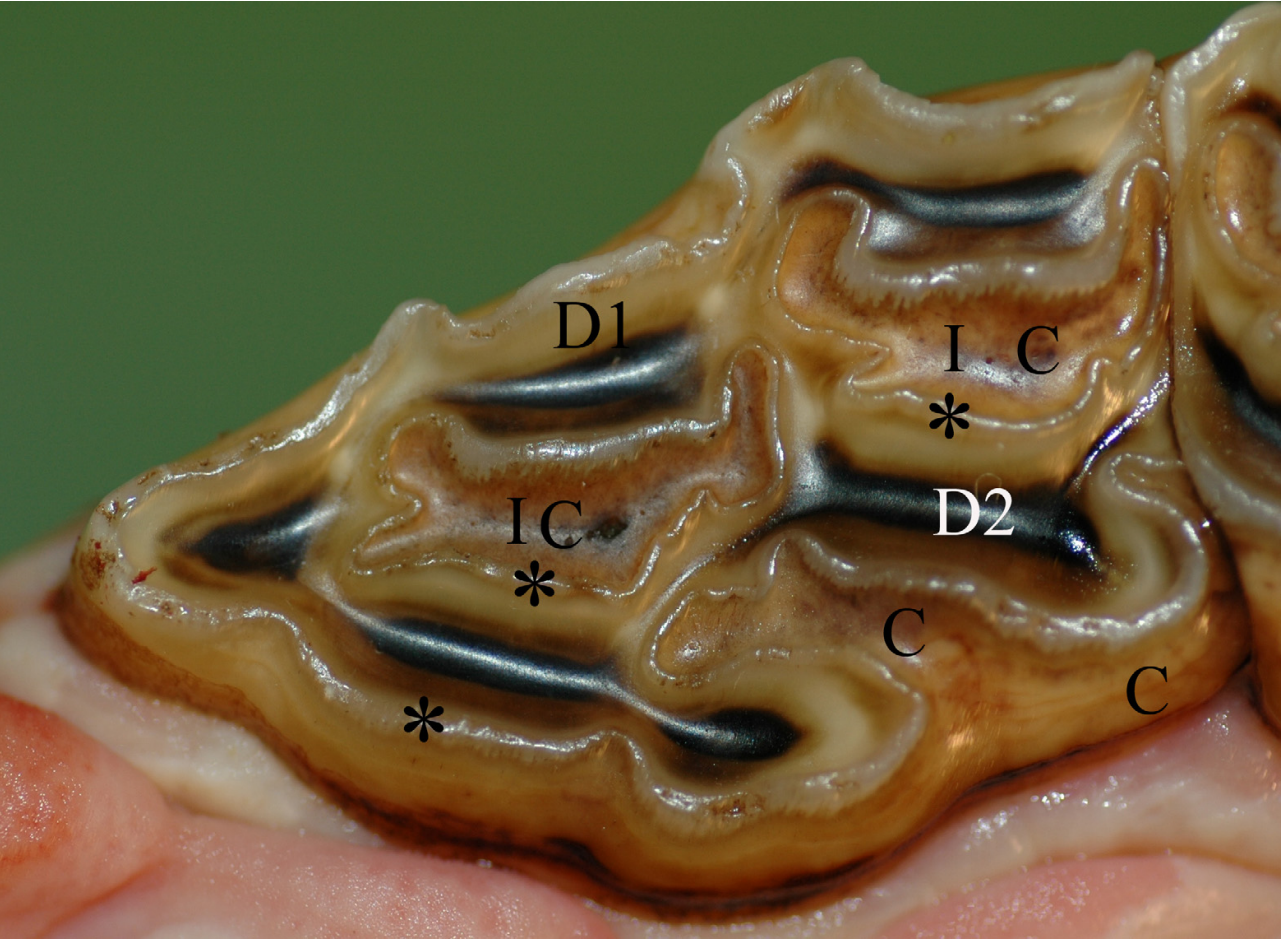
**Normal permanent maxillary P2**. Photograph of a P2 occlusal surface. C = cement (light brown), D1 = primary dentin (white/yellowish), D2 = secondary dentin overlying pulp horn (dark brown). * = enamel (visible as a winding ridge). I = infundibulum, (A cone shaped invagination from the occlusal surface of the tooth. The invagination is lined with enamel and filled with cementum (C) to different degrees).^8^

The development of dental caries in humans has been discussed in terms of an interaction between three main factors: bacteria, substrate, and teeth [1]. Owing to their ability to produce extracellular polysaccharides (polyglucans) from sucrose, certain bacterial species, e.g. streptococci, can adhere more easily to the tooth surface [2]. Members of the group of mutans streptococci (e.g. *S. mutans *and *S. sobrinus*) are unique in this sense, since their polyglucans are more water insoluble and become sticky when produced in dental plaque [1]. Tooth defects in the form of small fissures or enamel cracks, on the occlusal surface facilitate bacterial colonisation. In a favourable environment, such as the presence of abundant sugars within a tooth fissure, these bacteria produce lactic acids [3,4] in a manner that decreases the pH below the critical levels for demineralisation of cement, (pH < 6.7) [1] and enamel (pH ~ 5.5) [5,6]. A decrease in pH appears to cause very similar damage in human and equine enamels [7]. The subsequent removal of the tooth matrix by proteolysis leads to the so-called cavity of decay. The equine premolar tooth differs from its human counterpart in that it expresses all three types of dental hard tissue on its occlusal surface (Fig. 1). Necrosis of infundibular cementum, infundibular cemental hypoplasia, and micro fractures have all been suggested as factors that predispose carious lesions in equine teeth [8].

Honma *et al*. [9] classified tooth decay on a 5 grade scale, and the definitions of the different levels of this scale were modified by Dacre [10]when describing the severity of infundibular decay. When including all enamel, dentin and cement defects in the definition of dental caries, Honma *et al*. [9] reported a caries incidence of up to 97% in abattoir skulls from mainly older horses. Examining 355 abattoir skulls, Wafa [11] reported that 29% of these skulls had caries-affected teeth. When excluding infundibular cemental hypoplasia from the diagnosis, Brigham and Duncanson [12] found caries in 6 out of 50 abattoir sculls. However, when defining caries as a progressive demineralisation including infundibular cementum, enamel, and dentin, the malady has been found to have a prevalence of approximately 1% in the living equine populations of Germany and Sweden, [13,14] quite often secondary to primary diseases such as tooth fractures and congenital dental defects. The fourth upper premolar (P4) [8] and the first upper molars (M1) [8,9,15] have been reported to be the most commonly affected teeth.

In Swedish equine dental practices, the occurrence of carious lesions in the first permanent premolar teeth (P2) of the upper jaw as a primary disorder has empirically been found to have increased during the last 10 years. A previously unknown bacterium, *Streptococcus devriesei *(CCUG 47155^T^), has recently been isolated from such carious lesions [16]. This bacterium is related to *S. mutans *and appears to share its capability of adhering to the tooth surface. When given the right substrate, it produces copious extracellular polysaccharides that provide the bacteria with a favourable environment for multiplication. Like the human mutans streptococci [1]*S. devriesei *produces acid when fermenting sugars such as mannitol, sorbitol, raffinose, inulin and melibiose [16]. Thus, since it is capable of lowering the pH at the site of colonisation, it also shares the ability of *S. mutans *to dissolve the hydroxylapatite crystals in the dental hard tissues.

Prompted by the above observations, the purpose of this study was to test the hypothesis that *S. devriesei *is present in all carious upper P2 teeth in Swedish horses.

We also determined whether horses with such lesions had this type of bacteria on the occlusive surface of healthy premolar teeth.

## Methods

**Figure 2:**
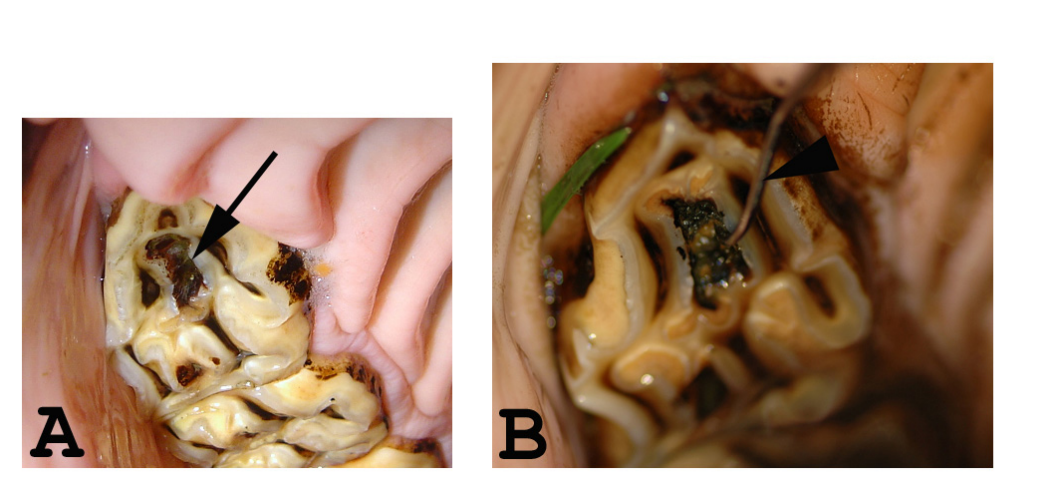
**P2 caries**. A and B: Intra-oral photographs of caries in the rostral infundibulum of a maxillary P2. A) Black arrow indicates a carious lesion. B) An investigation probe inserted into a carious lesion.

A total of 117 horses and ponies that had attended either the equine clinic at the Swedish University of Agricultural Sciences or the animal dental clinic in Söderköping, Sweden, for an oral examination as a part of a normal health check-up or after showing symptoms of an oral disorder were included in the study. Carious lesions were defined in this study as progressive decalcification and destruction of the cementum, enamel, and dentin in the infundibulum of the permanent P2 in the upper jaw (Fig. 2). Using the scale of Dacre, [10] this corresponds to grade 3 infundibular caries; that is, clinically the enamel ridge of the infundibulum is completely or partly missing and the decay feels sticky when an investigation probe is inserted into it.

Samples for bacteriological analyses were collected from P2 carious lesions in 27 animals between 1990 and 1999 and from 50 horses with P2 caries and 40 control animals with normal P2 occlusal surfaces after 2002. All caries lesions comprised the anterior infundibulum. All patients were examined and sampled by the same clinician (Dr. T. Lundström). All horses with caries had shown clinical signs such as unwillingness to eat and discomfort when ridden. None of the animals had been treated with any drugs or with antibiotics for 14 days and 6 months prior to sampling, respectively. Further, the controls had no diagnosis of general disease, nor had they been treated with NSAIDs or been examined or treated in the oral cavity for 6 months prior to sampling.

**Figure 3:**
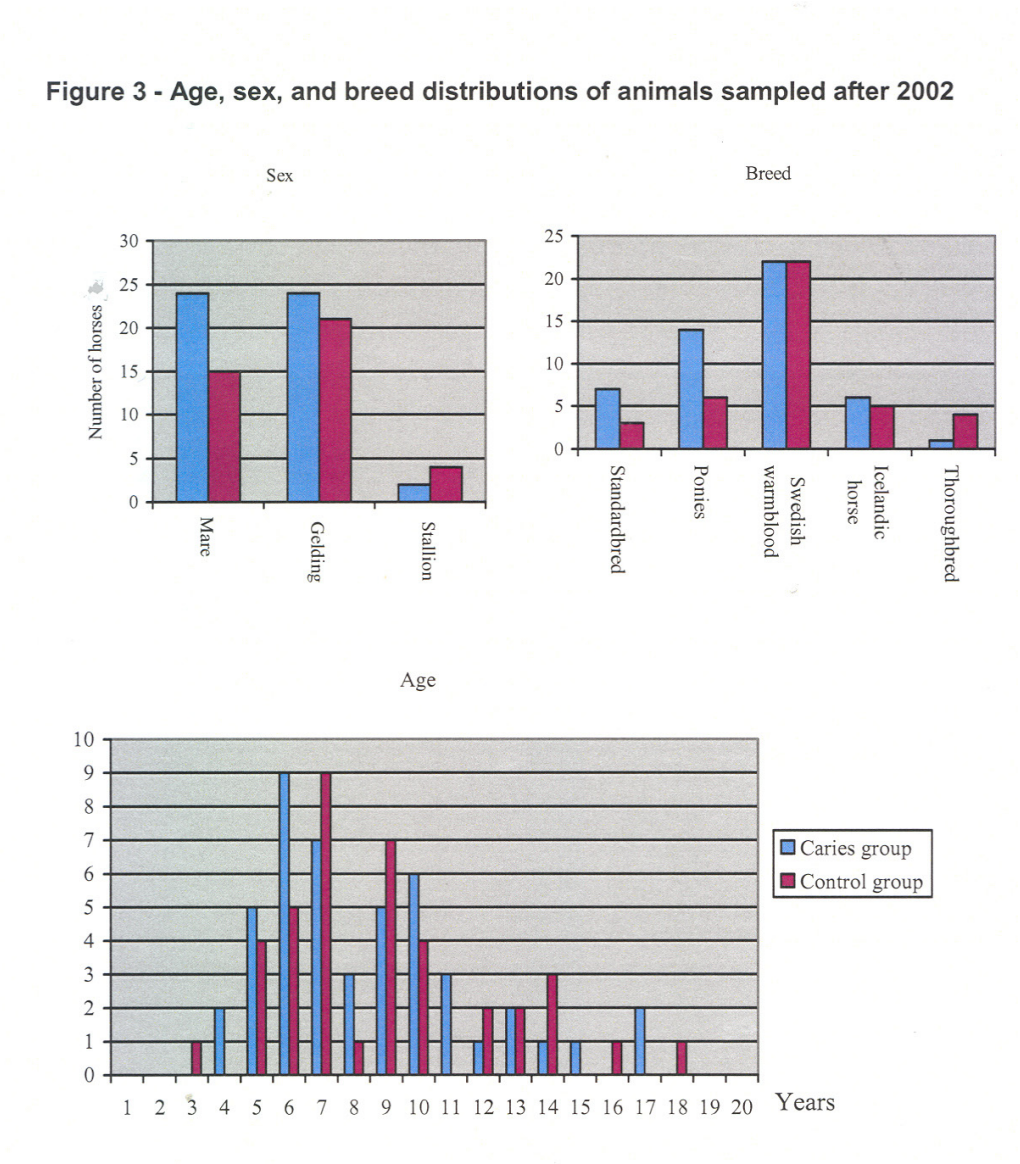
Age, sex, and breed distributions of animals sampled after 2002.

The age, sex, and breed distributions of animals sampled after 2002 are shown in Fig. 3. The animals sampled between 1990 and 1999 were between 7 and 17 years old (mean, 11 years) and consisted of 10 mares, 16 geldings, and 1 stallion. Four of them were ponies, 5 were Icelandic horses, 10 were Swedish warmbloods, and 8 were standardbred trotters.

### Sampling

**Figure 4:**
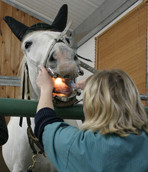
**Sampling procedure**. Sedated horse resting its head on a support bar during a dental examination.

**Figure 5:**
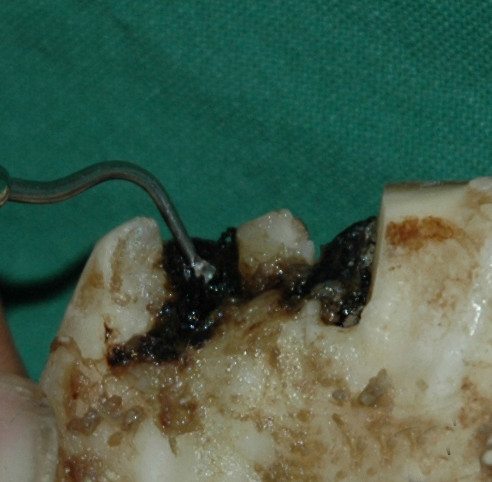
**Sampling procedure**. Dental excavator^3 ^inserted into a carious lesion.

**Figure 6:**
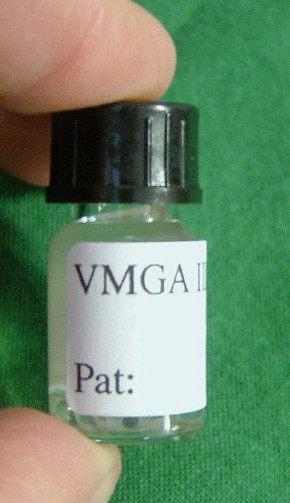
**Sampling procedure**. VMGA III^4 ^bacterial transport medium.

Each horse was given a sedative, detomidine (Domosedan^® ^Orion Pharma AB, Animal Health, Sollentuna, Sweden) at 10 μg/kg body weight intravenously, and its head was rested on a support to facilitate a dental examination (Fig. 4). A Haussmann oral speculum (Globus Sport AB, Karlskrona, Sweden) was attached and the oral cavity was rinsed with tap water of drinking quality. Samples were then collected by means of a sterile dental excavator (no.2 Straumann, Basel, Switzerland) that was inserted into the anterior infundibulum of caries-affected teeth (Fig. 5). The debris caught with the excavator was transferred to a bottle containing 3.3 ml of VMGA III (Viability Medium, Göteborg, Anaerobically prepared III, Sahlgrenska Academy, Göteborg, Sweden) transport medium (Fig. 6). VMGA III is a special transport medium for human oral bacteria, including streptococci [17]. The anterior infundibulum of the left or right P2 of the upper jaw of control horses was sampled randomly in the same manner. In control horses with a closed infundibulum, the sample was taken from the occlusal surface at the site of the infundibulum. Further, as an internal control, samples were taken from the second permanent premolar (P3) in the lower jaw on the opposite side in all control horses and in 30 of the animals with carious lesions.

The samples were sent the same day by mail to the Laboratory for Oral Microbiology, Faculty of Odontology, Sahlgrenska Academy at Göteborg University, Sweden, for analysis. The samples were all processed at the laboratory within 24 hours of the sampling procedure by a standard method for analysing the bacterial composition in dental caries, with special reference to *S. mutans *and related species, using a selective Mitis Salivarius-Bacitracin (MSB) agar [18]. The samples were made up in a 10-fold dilution series and 0.1 ml from each dilution step was inoculated onto an MSB agar plate and incubated for 3 days at 37°C in an atmosphere with 10% CO_2 _in N_2_. The appearance and numbers of colony forming units (CFU) of *S. devriesei *were determined visually according to standard procedures [19]. In accordance with these standard procedures, the total number of bacteria, CFU per ml VMGAIII, i.e. including all types of bacteria collected, was estimated in each sample and the results were categorised as 0, <100 000, 100 000 – 1 million, > 1 million CFU/ml. The isolates were characterised biochemically, using a commercially available kit (API Rapid ID32 Strep, API System, Biomerieux, Marcy 1Étoile, France) according to the manufacturer's instructions. The bacteria isolated from the carious lesions of the first six horses sampled during 2002 were also characterised taxonomically. The result of this characterisation has been presented elsewhere [16].

Fisher's exact test was used for statistical evaluation.

This project was approved by the Uppsala Animal Ethics Committee, diary no. C 231/4.

## Results

**Figure 7:**
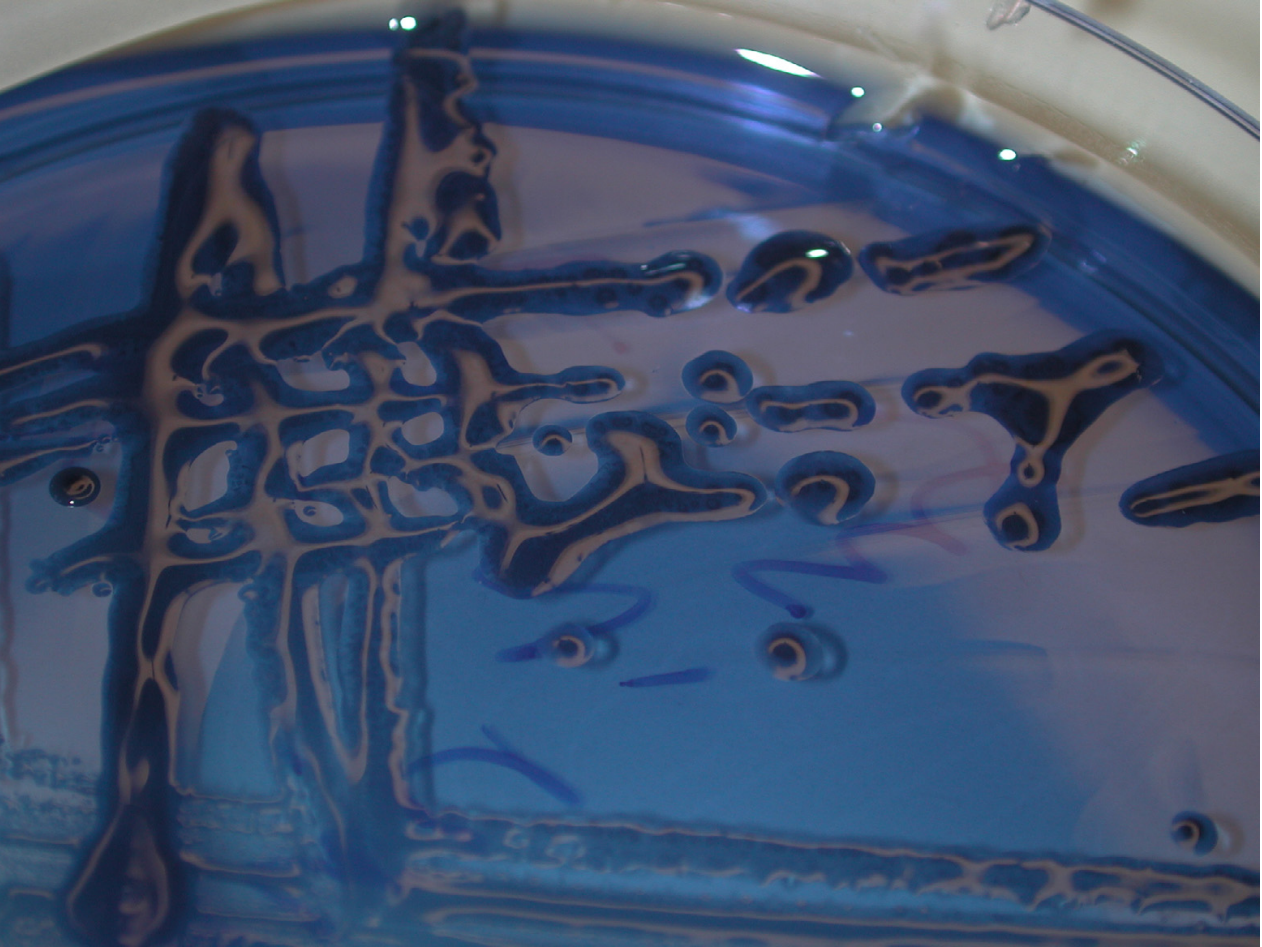
***Streptococcus devriesei *growing on MSB agar**. The extra cellular polysaccharides are evident as a transparent slime around the dark blue bacterial colonies.

There was no growth of *S. mutans*-like bacteria in samples taken from carious lesions during the years 1990 – 1999. On the contrary, the results of incubation on MSB agar and biochemical characterisation showed that all samples taken from carious lesions after 2002 were positive for the *S. mutans*-like strain, *S. devriesei*. Of these 50 animals with caries, 24 had carious lesions bilaterally in P2s of the upper jaw. In 20 of the 30 animals with caries sampled as an internal control, *S. devriesei *was also found in samples from the occlusal surface of the mandibular P3 on the opposite side (Table 1). The presence of *S. devriesei *colonies was significantly smaller in the control animals than in the samples taken from horses and ponies with carious lesions (p < 0.0001, 3 df). Among the control animals, four were positive for *S. devriesei *at the P2 position and of these horses three also had positive samples taken at the internal control site. The samples from the remaining 36 control animals were all negative for *S. mutans*-like bacteria. On the MSB agar plate, the *S. devriesei *isolates showed a copious production of polysaccharides (Fig. 7).

**Table 1:** Presence of bacteria in samples taken after 2002

Approximate number of bacteria (CFU/ml VMGAIII)	Animals with caries, P2 samples (*n *= 50)	Animals with caries, internal control (*n *= 30)	Control animals, P2 samples (n = 40)	Control animals internal control (*n *= 40)
0	0	10	36	37
< 100 000	6	7	4	3
100 000 to 1 million	11	5	0	0
> 1 million	33	8	0	0

Compared to control animals, *S. devriesei *was a significantly more common finding in horses with carious (*p *<*0.0001*, *3 df*). *S. devriesei *was significantly less prevalent (*p *< 0.0001), in positive control animals than in internal control samples from animals with caries.

## Discussion

Carious lesions are reported to mainly occur in cheek teeth of the upper jaw [20]. The gross anatomy differs between the upper and lower jaws with respect to the infundibula, which are absent in the mandibular cheek teeth. The infundibula in upper cheek teeth are usually incompletely filled by infundibular cementum and both Baker [8] and Kilic *et al*. [21] reported a high incidence of severe caries in this area. The incomplete filling with cementum could of course be looked upon as a typical predilection site, that is a damaged or incomplete tooth surface, where bacteria can easily adhere and colonise compared to the intact surface. Baker [8] suggested that Honma *et al*. [9] misnamed cemental hypoplasia as a carious lesion. However, it may be difficult to clinically define the difference between hypoplasia and caries of the cementum. In our experience, the caries definition used in the present study, – a progressive decalcification and destruction of the cementum, enamel and dentin in the infundibulum -, is more practical for clinical use.

All *S. mutans*-like bacteria from P2 carious lesions identified on the MSB agar showed the same pattern of growth and biochemical characteristics as the bacteria in the samples from the first six horses sampled in 2002, which were described by Collins *et al*.,[16] and we therefore suggest that they belong to the same bacterial species.

The aetiology of caries in horses is not known in detail but it is reasonable to regard it as multifactorial as is the case in humans. As mentioned above, in bacteria are regarded as one of the main causal factors for caries in humans. Bacteria that provided fermentable carbohydrates, sugars in particular, can colonise the oral cavity in contact with dental tissues. The present study shows that *S. devriesei *is associated with caries in P2 in Swedish horses. *S. mutans *and *S. devriesei *may have the same capability to adhere to the tooth surface, and given the right substrate e.g. sugars, they produce copious extracellular polysaccharides (Fig. 7) that provide the bacteria with a favourable environment for multiplication. Moreover, the fermentation of *S. devriesei *can lower the pH to the critical level at which dental tissue is demineralised, resulting in a caries lesion.

However, our result does not clarify the causal relationship between *S. devriesei *and dental decay. It can not be ruled out that the bacteria subsequently invade and colonise the lesion on account of a favourable environment in the caries cavity. We also found the bacteria, in four of the control animals, although in a much smaller number, which indicates that it can be present in the oral cavity of normal horses. Nevertheless, these horses may have been in the early stage of caries development without clinically visible demineralisation, or they may have been in the risk zone for developing carious lesions.

*S. mutans*-like bacterial isolates was not found in samples taken from P2 carious lesions between 1990 and 1999 even though all the samples analysed in this study were collected in the same way and analysed at the same laboratory using the same methods. This makes it likely that *S. devriesei *was introduced into the Swedish Equidae family after 2000. Nevertheless, it cannot be ruled out that an unidentified factor, in the sample handling and processing, influenced the results from the 1990s.

The numbers of horses and ponies have increased significantly in Sweden during the last 15 years, with this increase having been greatest around the major cities, and hence many horses live in urban surroundings with their foodstuff produced elsewhere. Within the same period of time, many new commercial equine food compositions have been introduced on the market, many of them being all-in-one feeds in the form of pellets, textured feeds, extruded nuggets and fortified hay cubes. In our experience, several of these products become sticky during consumption and hence adhere to the teeth surfaces. Whether any of these food compositions may be associated with P2 caries remains to be investigated. Future investigations should determine whether the composition of feed differed between the caries and control groups used in the present study. Saliva parameters should also be investigated in greater detail in horses, since we know that in humans the secretion rate (at rest and stimulated), pH, buffer capacity and chemical composition of saliva greatly influence the risk of developing caries [22].

## Conclusion

We suggest that *S. devriesei *forms part of the normal equine oral bacterial flora and that under certain conditions it colonises sites such as the infundibulum of the cheek teeth or traumatised dental hard tissues. It is obvious that such colonisation can be fatal for dental tissue. Further research into the normal function of saliva, food and immunological factors is needed to fully elucidate the aetiology of caries disease in horses.

## Competing interests

The author(s) declare that they have no competing interests.

## Authors' contributions

TL collected the samples, drafted the manuscript and participated in the design of the study, laboratory analysis and analysis of data. GD carried out the laboratory analysis and participated in the design of the study, analysis of data and drafting the manuscript. OW conceived the study, and participated in its design and coordination, analysis of data and helped to draft the manuscript. All authors read and approved the final manuscript
